# Concern about the Spread of COVID-19 in Regular Basic Education Teachers When Returning to Face-to-Face Classes

**DOI:** 10.3390/bs13040346

**Published:** 2023-04-20

**Authors:** Edwin Gustavo Estrada-Araoz, Judith Annie Bautista-Quispe, Zaida Esther Callata-Gallegos, Ronald Raul Arce-Coaquira, Yony Abelardo Quispe-Mamani, Percy Samuel Yabar-Miranda, Yolanda Paredes-Valverde, Rosel Quispe-Herrera

**Affiliations:** 1 Facultad de Educación, Universidad Nacional Amazónica de Madre de Dios, Puerto Maldonado 17001, Peru; 2 Facultad de Educación, Universidad Nacional del Altiplano, Puno 21001, Peru; jbautistaq@unap.edu.pe (J.A.B.-Q.); yquispe@unap.edu.pe (Y.A.Q.-M.); p.yabar@unap.edu.pe (P.S.Y.-M.); 3 Escuela Profesional de Educación Primaria, Universidad Nacional del Altiplano, Puno 21001, Peru; zaidacallata@unap.edu.pe; 4 Escuela Profesional de Gestión Pública y Desarrollo Social, Universidad Nacional de Moquegua, Moquegua 18001, Peru; rarcec@unam.edu.pe; 5 Facultad de Ecoturismo, Universidad Nacional Amazónica de Madre de Dios, Puerto Maldonado 17001, Peru; yparedes@unamad.edu.pe; 6 Facultad de Ingeniería, Universidad Nacional Amazónica de Madre de Dios, Puerto Maldonado 17001, Peru; rherrera@unamad.edu.pe

**Keywords:** mental health, COVID-19, contagion, post-pandemic, teachers, regular basic education

## Abstract

During the health emergency caused by COVID-19, a series of sensations such as fear, stress, and concern about contracting the virus were developed. Despite the fact that in recent months infection rates have been significantly reduced due to vaccination campaigns, the return of teachers to face-to-face classes established in Peru from April 2022 has increased once again the fear that contagion levels could grow. Therefore, the objective was to analyze the concern of regular basic education teachers about the spread of COVID-19 when returning to face-to-face classes. A quantitative investigation was carried out; the research design was observational and the type of study was descriptive cross-sectional. The sample was made up of 648 teachers who responded to the Scale of Concern for the Contagion of COVID-19, an instrument with adequate psychometric properties. The results show that 43.8% of teachers had moderate levels of concern about the spread of COVID-19, 38.7% had low levels, and 17.5% had high levels. Teachers reported most recurrent concerns about some risks in educational institutions and the fear of spreading COVID-19 to their relatives or people with whom they lived. On the other hand, it was found that some sociodemographic, occupational, and medical variables were significantly associated with this concern (*p* < 0.05). Then, it was concluded that teachers had moderate levels of concern about the spread of COVID-19 when returning to face-to-face classes.

## 1. Introduction

On December 2019, the first outbreak of the coronavirus (COVID-19) was detected in the Chinese city of Wuhan [1]. The first symptoms of COVID-19 are mild; however, after the seventh day of infection, it could rapidly progress to acute respiratory distress syndrome [2]. Damage to the respiratory system is the main factor leading to the death of infected people. However, the infection can affect other organs, such as the heart, kidneys, and liver, as well as immune and circulatory cells [3]. The presence of COVID-19 generated great concern in society, which is understandable since people are concerned about their health and do not want to get the virus, which causes sequelae and a potential risk of death [4].

Due to the exponential increase in cases worldwide, the World Health Organization (WHO) categorized COVID-19 as a pandemic in March 2021, a decision that caused a series of repercussions on health, social, economic, cultural and, of course, educational fields [5]. In Peru, to face the health emergency, the government proposed a series of restrictive measures, such as mandatory social isolation, in order to prevent the virus from spreading further [6]. In the educational field, academic activities were interrupted in elementary and high schools. However, in order to not affect significantly the continuity of educational service, the migration of education took place, which went from being face-to-face to being strictly virtual [7,8]. This unusual educational reform caused additional uneasiness among the teachers since many of them were not prepared or used to teaching virtually [9]. In addition, the increase in workload and the need to develop their digital skills contributed to higher levels of stress and demotivation. On the other hand, remote work posed a challenge as they needed to have access to technological devices and a stable and fast internet connection, as well as appropriate furniture to deliver virtual classes [10].

In the first quarter of 2022, the number of infections and deaths related to COVID-19 decreased significantly worldwide, mainly due to vaccination campaigns [11]. Therefore, the Ministry of Education of Peru ordered that in April 2022, classes will once again be held face-to-face and blended, both in universities and educational institutions [12,13]. For that reason, various protocols were established in order to prevent educational institutions from becoming the source of the contagion. These include mandating that all management, teaching, administrative and auxiliary personnel have to be vaccinated with three doses of the vaccine against COVID-19, making signs to maintain adequate distance, both in the classroom and the courtyard of the educational institution, promoting the use of masks throughout the educational community, and in any event where there is suspicion of possible contagion, notifying the corresponding educational authorities [14].

However, when classes began, it was observed that many of the protocols were not fully compliant, since it was perceived that in many institutions there were deficiencies in signage and physical distance was not respected. Not all teachers and staff working in the institution had three doses of the vaccine and some students and parents used their masks incorrectly. This scenario could have increased the concerns of being exposed to many risk factors for the spread of COVID-19 among teachers.

Concerns related to a disease can be defined as an emotional response that people have to pain, which is relevant for its proper control and influences the adoption of behaviors that promote health [15]. In the research related to respiratory infectious diseases, it is observed that, during the early stages, when the characteristics, treatment, and prevention of death are uncertain, affective risk responses can better predict the presence of protective behaviors [16]. However, if the concern is not well controlled, it could lead to a series of mental health problems, mainly translated into anxiety, stress, and anguish, which would have an impact on the psychological well-being of teachers, as well as on their quality of life [17]. For this reason, it is necessary to know and understand the levels of concern that they have about the spread of the virus since the findings could provide evidence for future interventions.

In this regard, some research was carried out to analyze the concern of teachers derived from the reopening of educational institutions. In Japan, they reported that the level of concern of teachers was high since they taught face-to-face classes in classrooms where around 30 and 40 students studied, which increased the probability of contagion. At the same time, the preventive measures and guidelines arranged by the national government and local governments seemed to be insufficient to prevent the spread of the virus [18]. Likewise, in Peru, they concluded that teachers were characterized by showing moderate levels of concern about the spread of COVID-19 due to the fact that in educational institutions there were various risk factors, such as the presence of students and parents who did not correctly use masks and adequate hand washing was not carried out [14]. On the other hand, in Germany, they determined that teachers were anxious and concerned after returning to face-to-face classes and considered that they were more likely to become infected [19].

Therefore, the research problem was: What is the level of concern among regular basic education teachers regarding the spread of COVID-19 when returning to face-to-face classes? Thus, the objective of this research was to analyze the concern about the spread of COVID-19 in regular basic education teachers during the return to face-to-face classes.

## 2. Materials and Methods

### 2.1. Design

The research had a quantitative approach, and the data collection was carried out to answer the research questions using statistics. Regarding the design of the research, it was observational, since the variable concern for the spread of COVID-19 was not intentionally manipulated, but rather was observed as it occurred in its environment and then analyzed. Regarding the type of study, it was descriptive cross-sectional, since the properties and characteristics of the variable of the study were described and because the data collection process occurred in a single moment [20].

### 2.2. Participants

The sample consisted of 648 regular basic education teachers from ten public institutions in Cusco, Peru, an amount determined by a sample size determined by non-probabilistic convenience sampling. Teachers who did not agree to participate in the research and those who incompletely filled out the data collection instrument were excluded. According to Table 1, 51.2% of participants were male and 48.8% were female. Regarding age group, 33.2% were from 41 to 50 years old, 26.2% from 31 to 40 years old, 21.6% from 51 to 64 years old, and 19% from 21 to 30 years old. Regarding the educational level, 40.7% were teachers at elementary school, 36.4% at high school, and 22.8% at elementary childhood education. Concerning labor conditions, 53.1% had open-ended contracts and 46.9% had temporary ones. Regarding vaccination status, 41.5% received three doses, 28.9% two, 17.4% one dose, and 12.2% were not vaccinated. Concerning comorbidity, 63.6% did not have comorbidities and 36.4% did. Regarding COVID-19, 65.4% had not previously been infected and 34.6% had been infected.

### 2.3. Instruments

The survey technique was used for data collection, while the instrument implemented was the Scale of Concern for the Contagion of COVID-19. This scale was elaborated by Carranza et al. [21] and was originally focused on personnel working in the health sector. However, it was adapted to the educational sector by Estrada et al. [14] and consists of 8 items of a single factor, it is of the Likert type (always, sometimes, and never) and it was determined in previous investigations that it had an adequate level of content validity (Aiken’s V = 0.847) and reliability (α = 0.833).

### 2.4. Procedure

The data collection was carried out between the months of May and July, 2022. Thus, a meeting was settled with the directors and vice-directors of the targeted educational institutions with the objective of informing them about the purpose of the research and requesting their respective authorization. Afterward, the teachers were contacted via WhatsApp and a link was sent where they could access and develop the scale, which was structured in Google Forms and lasted approximately 10 min. Access to the application was closed once the 648 responses were obtained, the information obtained was exported to a Microsoft Excel file, and the qualification process was carried out considering the respective assessment scale.

### 2.5. Data Analysis

To carry out the statistical analysis, SPSS Software version 25 was used. The descriptive results were systematized in a figure and a table, while the inferential results were obtained through the non-parametric chi-square test (X^2^), which allowed us to know if the variable of concern about the spread of COVID-19 was significantly associated with the proposed sociodemographic, occupational, and medical variables.

### 2.6. Ethical Considerations

Regarding ethical considerations, the present research was conducted in accordance with the ethical principles defined by the Helsinki Declaration and it had the endorsement of the institutional ethics committee. Likewise, it should be noted that the teachers were informed about the purpose and nature of the research and provided their informed consent, ensuring the private, confidential, anonymous, and voluntary nature of their participation.

## 3. Results

**Figure 1:**
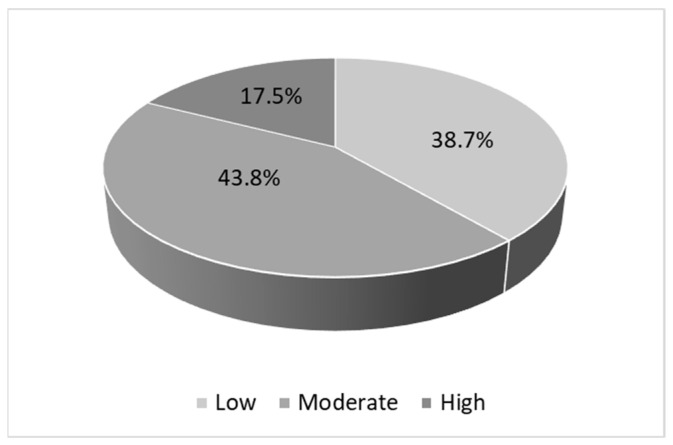
Level of concern of contagion of COVID-19.

According to Figure 1, the level of concern related to a disease can be defined as an emotional response that people have to pain, which is relevant for its proper control. A total of 43.8% of teachers were moderately concerned about a possible contagion of COVID-19 when returning to face-to-face classes, 38.7% had a low level of concern, and 17.5% had a high level of concern. The exposed result shows that teachers were characterized by being anxious and frightened because they considered that biosafety conditions and some risk factors could cause them to become infected with COVID-19 during the exercise of their duties in educational institutions. This would make the process of adaptation to face-to-face education more difficult and would affect their emotional well-being and satisfaction with the work they do.

Table 2 shows that the main concerns expressed by teachers were the possible spread of COVID-19, considering that there were some risks in educational institutions and the fear of spreading COVID-19 to their relatives or people with whom they lived. Some considered that they were most likely to get COVID-19 during their working hours and felt uncertainty because they considered COVID-19 to be an unpredictable disease.

The data presented in Table 3 show that concern about the spread of COVID-19 was significantly associated with the gender of teachers. In that sense, it can be said that the predominant level of concern in men was low, while in women, moderate. The data presented indicates that women had higher levels of concern than men about the spread of COVID-19.

Table 4 shows that concern about the spread of COVID-19 was significantly associated with the age group of the teachers. Under this premise, it can be seen that the predominant level of concern in teachers who were between 21 and 30 years old was low. However, teachers over 30 years of age were characterized by having a moderate level of concern. In addition, teachers whose ages fluctuated between 51 and 64 years felt more concerned than younger teachers.

According to Table 5, concern about the spread of COVID-19 was not significantly associated with the educational level of teachers. In this regard, it is observed that the predominant level of concern of teachers was moderate.

According to Table 6, concern about the spread of COVID-19 was significantly associated with the labor conditions of teachers. In this sense, despite the fact that both types of teachers were characterized by having a moderate level of concern, teachers with open-ended contracts reported a slightly higher level of concern compared to teachers with temporary contracts.

In Table 7, we can see that concern about the spread of COVID-19 was also significantly associated with the vaccination status of teachers. All teachers presented a moderate level of concern; however, teachers who had not been vaccinated or only received a single dose reported a slightly higher level of concern than other teachers.

As we can see in Table 8, concern about the spread of COVID-19 was significantly associated with the presence of comorbidities in teachers. In this regard, it is observed that the predominant level of concern for teachers with comorbidities was moderate. However, in the case of teachers who did not have comorbidities, it was low. Thus, it is possible to affirm that the teachers who declared comorbidities presented higher levels of concern than the teachers who did not report any comorbidities.

Finally, Table 9 shows that concern about the spread of COVID-19 was significantly associated with a previous infection in teachers. In this sense, we can see that in teachers who were previously infected and in those who were not infected, a moderate level of concern is predominated. However, there was a slightly higher level of concern among the teachers who had already been infected.

## 4. Discussion

During the health emergency caused by COVID-19, people expressed feelings of fear, stress and, above all, concern about getting the virus. Despite the fact that in recent months the contagion rates have been significantly reduced due to vaccination, the return of teachers to face-to-face classes established in Peru in April 2022 could have increased the fear that the contagion levels will increase. For this reason, in the present research, the concern of regular basic education teachers about the spread of COVID-19 when returning to face-to-face classes was analyzed.

It was found that the teachers were characterized by presenting moderate levels of concern about the spread of COVID-19. Among the main fears, they highlighted that there were some risks in educational institutions, and they feared infecting COVID-19 to their relatives or people with whom they lived with. This would impede the process of adaptation to face-to-face classes and would undoubtedly affect their emotional well-being and satisfaction with the work they performed.

This finding is consistent with what was reported in an investigation carried out in Japan, where they found that the concern of teachers derived from the reopening of educational institutions was moderate because they taught face-to-face classes in classrooms where between 30 and 40 students studied, which increased the probability of contagion [18]. Thus, it agrees with an investigation carried out in Peru, where they reported that teachers were characterized by presenting moderate levels of concern about the spread of COVID-19 due to the fact that in educational institutions there were various risk factors, such as the presence of students and parents who did not use the masks correctly and did not properly wash their hands [14]. On the other hand, it converges with a study carried out in Germany, in which they determined that teachers expressed moderate levels of anxiety and concerns when returning to face-to-face classes because they considered that they were more likely to become infected [19].

The European countries developed a proposal to reopen face-to-face educational service, which was based on three criteria, the first of which was epidemiological, ensuring that the spread of COVID-19 decreased. The second one was to ensure that the health system has sufficient equipment to care for the most serious cases, especially those that require hospitalization. The third one had the capacity to carry out large-scale detection tests to determine outbreaks [22]. In the same way, the WHO established that protocols must be implemented, which are described in a checklist to avoid an increase in the rate of infections in educational institutions [23]. The main actions are promoting hand hygiene, organizing classrooms respecting the distance between students, promoting the proper use of masks throughout the educational community, and respecting isolation procedures in the event that a student or teacher presents symptoms.

In Peru, the health authorities carried out several inspections in public educational institutions during the first quarter of 2022 to determine to what extent they complied with the protocols established by the Ministry of Education to return to face-to-face classes. For instance, many of the educational institutions did not comply with the prevention measures against COVID-19, such as signaling the entrance and exit of closed spaces, determining the entrance and exit doors of the educational premises, and having a station for hand washing and disinfection. Therefore, it was necessary to carry out actions to guarantee the quality of education and basic biosafety conditions for students and teaching staff [14].

A relevant finding shows that the gender of teachers was significantly associated with the level of concern about the spread of COVID-19. In this sense, it was determined that women were more concerned than men about possible contagion. This could be explained because they tend to suffer from internalizing disorders such as concern, depression, and anxiety in stressful situations, such as the pandemic or post-pandemic, and tend to externalize more emotional and physiological manifestations [24]. In the same way, it could also be explained by a cultural perspective, since they would feel more concerned about staying healthy to take care of their children.

There is some research that supports the findings described, which indicates that women are much more vulnerable than men to the impact of the pandemic on mental health. In Germany, an investigation was carried out and determined that women were more likely to be more concerned and anxious compared to men with regard to contracting COVID-19 [19]. Likewise, an investigation was carried out in Peru and found that women were characterized by having high levels of fear of contagion, while low and moderate levels predominated in men [25]. On the other hand, an investigation carried out in Mexico reported that women had a greater fear of being infected with COVID-19 than men, which was consistent with international reports on mental health that indicated that there was a higher prevalence of anxiety disorders and depression among women [26].

It was also found that the age group of teachers was significantly associated with the level of concern about the spread of COVID-19. This indicates that teachers who were over 51 years of age were more concerned about possible contagion compared to younger teachers, which would be explained by the fact that the death rate from COVID-19 was higher in older people. Age, therefore, generated higher levels of concern.

Actually, we could observe similar conclusions in an investigation carried out in Argentina, where they explored the attitudes and fears of the population regarding COVID-19 and determined that fear was greater in people between 55 and 59 years old [27]. Likewise, it is related to an investigation carried out in Peru, where it was searched to know the perception of fear in the Peruvian population during the COVID-19 pandemic and it was found that the perception of fear of possible contagion, hospitalization, and death due to the mentioned disease was higher in older people [28].

For instance, it was also found that the labor condition of teachers was significantly associated with the level of concern about the spread of COVID-19. In this sense, it was found that the teachers with temporary contracts were more concerned about a possible contagion than the ones with open-ended contracts and this is due to the fact that many of them (teachers with temporary contracts) work additionally, either in an educational institution or academy in order to increase their remuneration. Similarly, they are concerned about keeping their jobs and must think about where they will work next year. In addition, responsibilities fall on them, such as the coordination of cycles and grades, which on several occasions some of the teachers with open-ended contracts refuse to assume.

The described findings are consistent with what was reported by some research carried out in Peru [29,30] that also found higher levels of concern, stress, and mental disorders in teachers with temporary contracts compared to teachers with open-ended ones.

On the other hand, it was determined that the vaccination status of teachers was significantly associated with the level of concern they had regarding a possible new contagion of COVID-19. Actually, teachers who were not vaccinated or had only received one dose had higher levels of concern about possible contagion compared to those who had received two or three doses. As a matter of fact, having the full vaccine and booster doses reduces the transmission, morbidity, and mortality rates of the virus. However, not having any dose or only having one could increase the levels of concern, insecurity, and uncertainty because they are likely to be infected, present a severe COVID-19 case, and die [31].

Additionally, there is a considerable number of teachers who, due to the misinformation that exists about vaccines in some media and on social networks, chose not to get vaccinated. This aspect aggravated the situation because since the beginning of the pandemic, a large amount of false or inaccurate information was disseminated regarding the very existence of COVID-19, its origin, relevance, forms of contagion, prevention measures, and treatment [32]. In this sense, it is imperative to promote and develop scientific thinking, objectivity, and evidence-based beliefs to avoid misinformation, the proliferation of conspiracy theories, and the risks they imply, such as the difficulty of installing prevention measures against the spread of the virus, the use of ineffective or dangerous treatments, and the generation of more anxiety than already exists due to the pandemic [33].

It was also determined that the presence of comorbidities was significantly associated with the level of concern they had regarding a possible new contagion of COVID-19, that is, teachers who had two or more diseases at the same time had slightly higher levels of concern about possible contagion compared to teachers who did not have comorbidities. The scientific literature indicates that comorbidities increase lethality and the risk of hospitalization and morbidity from COVID-19, the main ones being renal and cardiovascular diseases, arterial hypertension, diabetes mellitus, immunodeficiencies, chronic respiratory diseases, and chronic liver diseases [34].

The finding described in the present research agrees with that one reported on an investigation carried out in Argentina, where they determined that there was a greater fear of contagion from COVID-19 in people who presented risk or vulnerability factors. For example, those who suffered from diabetes and cardiovascular diseases [27]. Likewise, it agrees with the results of a study carried out in Peru, where they inquired about what factors were associated with the development of depression, anxiety, fear, and stress in the context of the COVID-19 pandemic and determined that being single, having contracted the virus, and having comorbidities were significantly associated with said emotional problems [35].

Finally, it was determined that having previously been infected with COVID-19 was significantly associated with higher levels of concern about a possible new contagion of the virus, especially since many people who already had COVID-19 were symptomatic. In fact, they had several symptoms and were even hospitalized, which makes them fear reinfection and health complications.

COVID-19 has infected millions of people around the world, and many countries have reported large numbers of deaths. People who have recovered from COVID-19 were thought to acquire a robust immune response and develop protective immunity. However, since the first documented case of COVID-19 reinfection in August 2020, there have been many cases of reinfection. There are cases that lack genomic data for the two infections, and it is not clear if they were caused by different strains [36]. Therefore, people, regardless of their prior infection history, should continue to participate in mitigating the spread of infection.

This research addresses an important issue that can affect the personal well-being and development of teachers, an aspect that enhances its relevance. However, it is necessary to specify some limitations. Firstly, the sample size is relatively small, and it is also homogeneous, which implies caution when interpreting the results. Secondly, the findings are based entirely on data obtained from a self-administered instrument, which could have led to subjective judgments by the participants. In this order of ideas, it is expected that further research will expand the size of the sample, including teachers, both from urban and rural areas, as well as from different sociocultural characteristics, and use other data collection instruments that complement those that were applied to give greater objectivity to the aforementioned process.

## 5. Conclusions

The health emergency that has arisen since 2020 has been an inflection point in the history of humanity due to the repercussions that are caused from every point of view. However, returning to face-to-face education after more than two years of virtual education is causing concerns among teachers due to possible infections and reinfections that could arise despite the fact that the Ministry of Education of Peru established protocols to safeguard the health of the educational community.

In the present investigation, it was concluded that the teachers were characterized by presenting moderate levels of concern about the spread of COVID-19 when returning to face-to-face classes. Among the main fears were considering that there were some risks in educational institutions and the fear of infecting their relatives or people with whom they lived with COVID-19. Likewise, it was established that female teachers, who were over 51 years old, who had temporary contracts, who were not vaccinated or had only one dose, who had comorbidities and had previously been infected with COVID-19 had slightly higher levels of concern of the contagion compared to the other contrast groups.

In conclusion, it is suggested that the Ministry of Education and the Ministry of Health, as well as their decentralized instances, should constantly verify compliance with biosafety protocols in educational institutions. In the same way, it is necessary for teachers to include, within their curricular planning topics, strategies related to preventive measures to avoid a new spread of COVID-19.

## Figures and Tables

**Table 1:** Sociodemographic, occupational, and medical characteristics of the sample.

Sociodemographic, Occupational, and Medical Characteristics		n = 648	%
Gender	Male	316	48.8
	Female	332	51.2
Age group	From 21 to 30 years old	123	19.0
	From 31 to 40 years old	170	26.2
	From 41 to 50 years old	215	33.2
	From 51 to 64 years old	140	21.6
Educational level	Early childhood education	148	22.8
	Elementary	264	40.7
	High school	236	36.4
Labor condition	Temporary contract	304	46.9
	Open-ended contract	344	53.1
Vaccination status	Unvaccinated	79	12.2
	Vaccinated: 1 dose	113	17.4
	Vaccinated: 2 doses	187	28.9
	Vaccinated: 3 doses	269	41.5
Comorbidity	Yes	236	36.4
	No	412	63.6
Previous contagion of COVID-19	Yes	224	34.6
	No	424	65.4

**Table 2:** Answers to the questions related to the Scale of Concern of Contagion of COVID-19.

Items	Always		Sometimes		Never	
	n	%	n	%	n	%
I am concerned about the possibility of getting COVID-19 during my working hours.	140	21.6	303	46.8	205	31.6
Thinking about the possibility of getting COVID-19, while doing my job, anguishes me.	80	12.3	291	44.9	277	42.7
Thinking about the possibility of getting COVID-19 on the job does not let me sleep well.	72	11.1	225	34.7	351	54.2
Thinking about the possibility of getting COVID-19 affects my ability to carry out my work and personal activities.	103	15.9	244	37.7	301	46.5
When I leave work, I am concerned about spreading COVID-19 to family members or people I live with.	143	22.1	306	47.2	199	30.7
COVID-19 causes me uncertainty because it is an unpredictable disease.	95	14.7	305	47.1	248	38.3
In my workplace, there are many risks that make me concerned about being infected with COVID-19.	156	24.1	317	48.9	175	27.0
Despite putting biosecurity measures into practice to avoid getting COVID-19, I continue to be concerned.	125	19.3	272	42.0	251	38.7

**Table 3:** Association between concern about the spread of COVID-19 and the gender of the participants.

Variables		Concern about Getting COVID-19			*p*
		High	Moderate	Low	
Gender	Male	39 (12.3%)	124 (39.2%)	153 (48.4%)	0.000 *
	Female	74 (22.3%)	160 (48.2%)	98 (29.5%)	

* Statistically significant association.

**Table 4:** Association between concern about the spread of COVID-19 and the age group of the participants.

Variables		Concern about Getting COVID-19			*p*
		High	Moderate	Low	
Age group	From 21 to 30 years old	11 (8.9%)	28 (22.8%)	84 (68.3%)	0.038 *
	From 31 to 40 years old	25 (14.7%)	76 (44.7%)	69 (40.6%)	
	From 41 to 50 years old	42 (19.5%)	108 (50.2%)	65 (30.2%)	
	From 51 to 64 years old	35 (25.0%)	72 (51.4%)	33 (23.6%)	

* Statistically significant association.

**Table 5:** Association between concern about the spread of COVID-19 and educational level.

Variables		Concern about Getting COVID-19			*p*
		High	Moderate	Low	
Educational level	Early Childhood Education	25 (16.9%)	64 (43.2%)	59 (39.9%)	0.067
	Elementary	46 (17.4%)	119 (45.1%)	99 (37.5%)	
	High School	42 (17.8%)	101 (42.8%)	93 (39.4%)	

**Table 6:** Association between concern about the spread of COVID-19 and the labor condition of the participants.

Variables		Concern about Getting COVID-19			*p*
		High	Moderate	Low	
Labor condition	Temporary contract	71 (23.4%)	126 (41.4%)	107 (35.2%)	0.033 *
	Open-ended contract	42 (12.2%)	158 (45.9%)	144 (41.9%)	

* Statistically significant association.

**Table 7:** Association between concern about the spread of COVID-19 and the vaccination status of the participants.

Variables		Concern about Getting COVID-19			*p*
		High	Moderate	Low	
Vaccination status	Unvaccinated	19 (24.1%)	35 (44.3%)	25 (31.6%)	0.042 *
	Vaccinated: 1 dose	28 (24.8%)	49 (43.4%)	36 (31.8%)	
	Vaccinated: 2 doses	25 (13.4%)	83 (44.4%)	79 (42.2%)	
	Vaccinated: 3 doses	41 (15.2%)	117 (43.5%)	111 (41.3%)	

* Statistically significant association.

**Table 8:** Association between concern about the spread of COVID-19 and the comorbidity of participants.

Variables		Concern about Getting COVID-19			*p*
		High	Moderate	Low	
Comorbidity	Yes	62 (26.3%)	121 (51.3%)	53 (22.4%)	0.006 *
	No	51 (12.4%)	163 (39.6%)	198 (48.0%)	

* Statistically significant association.

**Table 9:** Association between concern about the spread of COVID-19 and previous contagion of participants.

Variables		Concern about Getting COVID-19			*p*
		High	Moderate	Low	
Have you already got COVID-19?	Yes	43 (19.2%)	100 (44.6%)	81 (36.2%)	0.040 *
	No	70 (16.5%)	184 (43.4%)	170 (40.1%)	

* Statistically significant association.

## Data Availability

Not applicable.
